# Priming of Anti-tumor Immune Mechanisms by Radiotherapy Is Augmented by Inhibition of Heat Shock Protein 90

**DOI:** 10.3389/fonc.2020.01668

**Published:** 2020-08-27

**Authors:** Anne Ernst, Roman Hennel, Julia Krombach, Heidi Kapfhammer, Nikko Brix, Gabriele Zuchtriegel, Bernd Uhl, Christoph A. Reichel, Benjamin Frey, Udo S. Gaipl, Nicolas Winssinger, Senji Shirasawa, Takehiko Sasazuki, Markus Sperandio, Claus Belka, Kirsten Lauber

**Affiliations:** ^1^Department of Radiation Oncology, University Hospital, LMU Munich, Munich, Germany; ^2^Department of Otorhinolaryngology, University Hospital, LMU Munich, Munich, Germany; ^3^Walter Brendel Center for Experimental Medicine, Faculty of Medicine, LMU Munich, Munich, Germany; ^4^Department of Radiation Oncology, Universitätsklinikum Erlangen, Friedrich-Alexander-University Erlangen-Nürnberg (FAU), Erlangen, Germany; ^5^Department of Organic Chemistry, NCCR Chemical Biology, University of Geneva, Geneva, Switzerland; ^6^Department of Cell Biology, Faculty of Medicine Fukuoka University, Fukuoka, Japan; ^7^Institute for Advanced Study, Kyushu University, Fukuoka, Japan; ^8^Institute of Cardiovascular Physiology and Pathophysiology, Biomedical Center, LMU Munich, Munich, Germany; ^9^German Cancer Consortium (DKTK), Partner Site Munich, Heidelberg, Germany

**Keywords:** HSP90 inhibition, radiotherapy, anti-tumor immunity, immune priming, colorectal cancer, cancer immunology, DAMPs, secondary necrosis

## Abstract

Radiotherapy is an essential part of multi-modal cancer therapy. Nevertheless, for certain cancer entities such as colorectal cancer (CRC) the indications of radiotherapy are limited due to anatomical peculiarities and high radiosensitivity of the surrounding normal tissue. The development of molecularly targeted, combined modality approaches may help to overcome these limitations. Preferably, such strategies should not only enhance radiation-induced tumor cell killing and the abrogation of tumor cell clonogenicity, but should also support the stimulation of anti-tumor immune mechanisms – a phenomenon which moved into the center of interest of preclinical and clinical research in radiation oncology within the last decade. The present study focuses on inhibition of heat shock protein 90 (HSP90) whose combination with radiotherapy has previously been reported to exhibit convincing therapeutic synergism in different preclinical cancer models. By employing *in vitro* and *in vivo* analyses, we examined if this therapeutic synergism also applies to the priming of anti-tumor immune mechanisms in model systems of CRC. Our results indicate that the combination of HSP90 inhibitor treatment and ionizing irradiation induced apoptosis in colorectal cancer cells with accelerated transit into secondary necrosis in a hyperactive Kras-dependent manner. During secondary necrosis, dying cancer cells released different classes of damage-associated molecular patterns (DAMPs) that stimulated migration and recruitment of monocytic cells *in vitro* and *in vivo*. Additionally, these dying cancer cell-derived DAMPs enforced the differentiation of a monocyte-derived antigen presenting cell (APC) phenotype which potently triggered the priming of allogeneic T cell responses *in vitro*. In summary, HSP90 inhibition – apart from its radiosensitizing potential – obviously enables and supports the initial steps of anti-tumor immune priming upon radiotherapy and thus represents a promising partner for combined modality approaches. The therapeutic performance of such strategies requires further in-depth analyses, especially for but not only limited to CRC.

## Introduction

Radiotherapy is a cornerstone of multi-modal cancer treatment. Its therapeutic efficacy is primarily considered to derive from direct tumor cell killing and the abrogation of tumor cell clonogenicity (1, 2). Additionally, there is growing evidence for a relevant contribution of the immune system to local as well as distant tumor control, and the concept of cancer *in situ* vaccination by radiotherapy is receiving increasing acceptance (3, 4). In this regard, the mode of cancer cell death induced by radiotherapy appears to be of fundamental importance. The priming of anti-tumor immune mechanisms has predominantly been observed in the context of necrotic forms of cell death due to the release of damage-associated molecular patterns (DAMPs) paralleled by the stimulation of an intra-tumoral type I interferon response (5, 6). Yet, the mode of irradiation-induced cell death varies considerably and depends on several factors, including the origin and genetic repertoire of the irradiated cell, the irradiation quality, the fractionation regimen, and the overall dose (4). With photon irradiation, higher single doses or strongly hypofractionated protocols, such as 3 × 8 Gy, seem to be beneficial for the stimulation of systemic anti-tumor immune mechanisms (7–10). We and others have shown that DAMPs released from irradiated, dying tumor cells stimulate the activation of endothelial cells and the recruitment of antigen presenting cells (APCs) which then crossprime CD8^+^ T cells in a type I interferon-dependent manner involving the cGAS/STING axis (8, 9, 11–14).

Despite its broad relevance for the treatment of other solid cancer entities, indications of radiotherapy in colorectal cancer (CRC) remain largely confined to malignancies of the rectum (15–17). The increased mobility of the colon and the resulting challenges of treatment volume definition and dose administration, as well as the high degree of radiosensitivity of the surrounding normal tissue limit the application of radiotherapy in colon cancer to high-risk cases receiving adjuvant fractionated (1.8–2 Gy per fraction) or neoadjuvant hypofractionated (5 Gy per fraction) radiotherapy alone or in combination with systemic chemotherapy, respectively (16, 17). Particularly for these high-risk cases it would be of relevant clinical interest to therapeutically exploit not only the induction of tumor cell death and abrogation of clonogenicity but also the radiotherapy-induced priming of anti-tumor immune mechanisms. To this end, various combined modality approaches with molecularly targeted agents are currently being explored, including inhibition of heat shock protein 90 (HSP90) (18). The chaperone HSP90 is frequently overexpressed in tumors due to high protein turnover and constitutively increased levels of proteotoxic stress (19). It contributes to maintaining the integrity, correct folding, and stability of diverse oncogene products (20, 21). Within the large substrate spectrum, many HSP90 client proteins belong to oncogenic signaling pathways and thus orchestrate the malignant phenotype (22, 23). Hence, interference with HSP90 function appears to be a promising strategy to target cancer cells *via* multiple axes, and several HSP90 inhibitors (HSP90i) showed encouraging preclinical results (24, 25). In contrast however, most clinical trials with HSP90i monotherapy failed due to poor therapeutic efficacy and an unfavorable spectrum of side effects, particularly in terms of hepatotoxicity (26). Nevertheless, since key regulators of the DNA damage response have been reported to be specifically sensitive to HSP90i treatment, even at low inhibitor concentrations, combinations of HSP90i with radio- and/or chemotherapy recently moved into focus. The superordinate aim of these approaches is to improve the therapeutic performance and at the same time reduce the required HSP90i doses and concurrent adverse effects (27–33). For preclinical models of CRC, the radiosensitizing potential of HSP90i treatment has already been demonstrated (34, 35). We have previously shown that the second generation HSP90i NW457 exhibits reduced hepatotoxicity and potently sensitizes CRC cells toward ionizing irradiation *in vitro* and *in vivo* by interfering with the DNA damage response (36–38). The underlying mechanisms of radiation-induced cell death in the presence of HSP90i are currently being dissected (39). However, the immunological potential of HSP90i-mediated radiosensitization has not yet been examined, although seminal data suggest that HSP90i may augment the stimulation of anti-tumor immune mechanisms (19). Accordingly, the present study was designed to elucidate the immunological aspects of HSP90 inhibition in combination with ionizing irradiation in model systems of CRC. We show that the combination of radiotherapy and HSP90i treatment induced apoptosis and accelerated transit into secondary necrosis in CRC cells with concomitant release of DAMPs. These DAMPs mediated migration and recruitment of monocytic cells *in vitro* and *in vivo* and enhanced the differentiation of a monocyte-derived APC phenotype which potently activated proliferation of allogeneic CD4^+^ and CD8^+^ T cells *in vitro*.

## Materials and Methods

### Cells, Animals, and Reagents

The human CRC cell lines HCT116 Kras^+/G13*D*^ Bax^+/+^ and HCT116 Kras^+/G13*D*^ Bax^–/–^ were kindly provided by B. Vogelstein (Baltimore, MD, United States) (40), and HCT116 Kras^+/–^ Bax^+/+^ cells (Hke3) were a generous gift from S. Shirasawa and T. Sasazuki (Fukuoka University, Japan) (41). Cells were cultured in McCoy’s full medium (McCoy’s 5A medium supplemented with 10% heat-inactivated fetal calf serum (FCS) [both from Thermo Fisher Scientific, Dreieich, Germany), 100 units/ml penicillin, and 0.1 mg/ml streptomycin (PS) (Lonza, Cologne, Germany)] at 37°C and 7.5% CO_2_. HCT8, HT29, and SW480 CRC cells and monocytic THP-1 cells were obtained from ATCC or DSMZ (Braunschweig, Germany) and were cultivated in DMEM full medium (DMEM (Thermo Fisher Scientific) supplemented with 10% FCS, 100 units/ml penicillin, and 0.1 mg/ml streptomycin (PS) for HCT8 and HT29 cells) at 37°C and 7.5% CO_2_ or RPMI 1640 full medium [RPMI 1640 medium (Thermo Fisher Scientific) supplemented with 10% FCS, 100 units/ml penicillin, 0.1 mg/ml streptomycin (PS), and 1% 4-(2-hydroxyethyl)-1-piperazineethanesulfonic acid (HEPES, Lonza) for SW480 and THP-1 cells] at 37°C and 5% CO_2_, respectively. Cell line authenticity was confirmed by short tandem repeat typing (service provided by the DSMZ), and absence of mycoplasma contamination was ensured by regular testing (MycoAlert Mycoplasma Detection Kit, Lonza).

Experiments with primary blood cells from healthy volunteers were approved by the ethics committee of the medical faculty of the LMU Munich and were performed upon written informed consent. Primary human monocytes were prepared from heparinized blood from healthy donors as described before (42) and cultured in X-Vivo full medium (X-Vivo 15 medium (Lonza) supplemented with 10% autologous serum (AS) and PS) at 37°C and 5% CO_2_. Monocytes were differentiated to dendritic cells with 20 ng/ml GM-CSF and 40 ng/ml IL-4 for up to 5 days (all cytokines from R&D Systems, Wiesbaden, Germany). Naïve T cells were isolated by Biocoll density gradient centrifugation (density 1.077 g/ml, Biochrom AG, Berlin, Germany) from heparinized blood of healthy donors followed by anti-CD3 magnetic bead separation (Miltenyi, Bergisch Gladbach, Germany) according to the manufacturer’s recommendations.

All *in vivo* analyses were performed in accordance with the Federation of European Laboratory Animal Science Associations (FELASA) guidelines and with the approval of local government authorities (*Regierung von Oberbayern*). Male C57BL/6 or Balb/c mice were purchased from Charles River (Sulzfeld, Germany). CX_3_CR-1^*GFP/+*^ C57BL/6 mice were generated as described previously and backcrossed for 6 to 10 generations to the C57BL/6 background (43). Mice used in the experiments were 10 to 13 weeks old and housed under standard conditions with access to food and water *ad libitum*.

The pochoxime-derived HSP90 inhibitor (HSP90i) NW457 (*epi*-pochoxime F; HSP90i) was described previously (44–46). A 10 mM stock solution was prepared in dimethyl sulfoxide (DMSO) and stored at -20°C as described (37, 38). Calcein-AM was purchased from Merck Millipore (Darmstadt, Germany), carboxyfluorescein diacetate succinimidyl ester (CFSE) from Thermo Fisher Scientific, uridine 5′-diphoshoglucose (UDP-glucose) from Abcam (Cambridge, United Kingdom), and IL-4, GM-CSF, TNF, SDF-1α, and WKYMVm (agonist of formyl-peptide receptors 1, 2, and 3) from R&D Systems. All other reagents were obtained from Sigma-Aldrich (Taufkirchen, Germany), if not stated otherwise.

### Radiation Treatment and Preparation of Cell-Free, Conditioned HCT116 Cell Culture Supernatants

Cells (0.9 × 10^6^ cells/well in a 6-well plate) were γ-irradiated with an RS225 X-ray cabinet (Xstrahl Ltd., Camberley, United Kingdom; 200 kV, 10 mA, Thoraeus filter, 1 Gy in 63 s) or with a Mueller RT-250 x-ray tube (200 kV, 10 mA, Thoraeus filter, 1 Gy in 1 min 52 s). Subsequently, full medium was replaced by serum-free medium supplemented with the indicated HSP90i concentrations. Cell-free supernatants were collected by centrifugation (10,000 × *g*, 5 min, 4°C) at the indicated time points after irradiation and stored at −80°C until further use.

### Analysis of DAMPs in HCT116 Cell Culture Supernatants

HSP70, high mobility group box 1 (HMGB1) and UTP levels in cell-free supernatants were measured by ELISA (HSP70, R&D Systems; HMGB1, IBL International, Hamburg, Germany; UTP, USCN, Wuhan, China). ATP levels were analyzed by luciferase tests (ATP Determination Kit, Thermo Fisher Scientific, Waltham, MA, United States) on a Synergy MX plate reader (BioTek, Bad Friedrichshall, Germany).

### Transwell Migration Assay

Transwell migration of calcein-labeled THP-1 cells toward cell-free supernatants or positive controls was allowed for 90 min with 96-well Multiscreen-MIC transwell chambers with 5 μm pore size (Merck Millipore, Darmstadt, Germany) as described before (42). Transmigrated THP-1 cells were collected by centrifugation, and transmigration was calculated from the calcein-fluorescence signal measured upon lysis on a Synergy MX plate reader (BioTek, Bad Friedrichshall, Germany) as percentage of total THP-1 cells applied.

For the biochemical characterization of monocyte attraction signals, cell-free supernatants harvested 48 h post treatment and ultracentrifuged at 42,000 × *g* (143 min, 4°C) were employed. Supernatants were subjected to heat-treatment at 90°C for 40 min, ultrafiltration with VivaSpin2 tubes with an MW cut-off of 10 kDa (Sartorius Stedim Biotech, Göttingen, Germany), or enzymatic digestion with active or heat-inactivated proteinase K (3 U/ml) or apyrase (50 mU/ml) at 30°C for 30 min (both from New England Biolabs, Frankfurt, Germany), respectively. Culture medium supplemented with either ATP (200 nM) or SDF-1α (200 ng/ml) served as control and was treated analogously to the cell culture supernatants. Migration stimulating capacity of the processed supernatants was assessed in THP-1 cell transwell migration assays and normalized to non-treated supernatants.

### Chemotaxis/Chemokinesis Assays

Chemotaxis and chemokinesis of primary human monocytes toward conditioned supernatants or apyrase digested-supernatants were analyzed with IBIDI μ-slide 2D-chemotaxis chambers (IBIDI, Munich, Germany) by live cell tracking for 3 h as described previously (42). Time-lapse video microscopy was performed on an AxioObserver Z1 inverted microscope with a heat stage (37°C, 5% CO_2_) at 5 × magnification. Cell tracking was performed with ImageJ (National Institutes of Health, Bethesda, MD, United States), and cell tracks (2.5 h time frame) were analyzed with the IBIDI chemotaxis and migration tool to determine accumulated distance, Euclidean distance (linear distance between the start and end position), and forward migration index (FMI, defined as the distance from start to endpoint in gradient direction divided by the total accumulated distance).

### Flow Cytometric Analyses of Apoptotic DNA Fragmentation, Plasma Membrane Integrity, and APC Surface Marker Expression

Flow cytometric measurements were performed on an LSRII cytometer (BD Biosciences, Heidelberg, Germany), and data were analyzed with FACSDiva (BD Biosciences) or FlowJo 7.6.5 Software (Tree Star Inc., Ashland, OR, United States), respectively.

Cells (4–8 × 10^4^ cells/well in 24-well plate) were irradiated with 0–5 Gy in the presence of 0–625 nM HSP90i. Cells without treatment were used as controls. Time course analyses of apoptosis and necrosis induction were analyzed by flow cytometry as described previously (38). For analyses of secondary necrosis and necroptosis, the poly-caspase inhibitor zVAD-fmk (50 μM; Bachem, Bubendorf, Switzerland) or the necroptosis inhibitor necrostatin-1 (50 μM; Enzo Life Sciences, Loerrach, Germany) were used in addition to irradiation and HSP90i treatment.

For APC surface marker expression analyses, primary human monocytes (0.8–2 × 10^5^ cells/well in 24-well plate) were cultured for 5 days in the presence of conditioned HCT116 culture supernatants (1 + 1 in X-Vivo full medium) with or without addition of GM-CSF (20 ng/ml) and IL-4 (40 ng/ml). 100 ng/ml TNF served as positive control. After 5 days, APCs were harvested, and stained for 20 min on ice with the following monoclonal antibodies in FACS stain buffer (BD Biosciences): anti-HLA-DR-Per-CP-Cy5.5, anti-CD86-A700, anti-CD80-PE, anti-CD40-PE-Cy5 or corresponding isotypes (all from BD Biosciences). Following two washing steps, cells were examined by flow cytometry. Expression levels of cell surface markers were calculated as median fluorescence intensities subtracted by corresponding isotype controls and are displayed as x-fold expression values compared to values obtained with HCT116 control supernatants.

### Phagocytosis Assays

Phagocytosis experiments were performed as described previously (11). In brief, PKH67-labeled primary human monocytes (10^5^ cells/well in a 24-well plate) were differentiated for 4 days into DCs by addition of GM-CSF (20 ng/ml) and IL-4 (40 ng/ml) and were then added to Hoechst-labeled HCT116 cells (ratio 1:2). Adherence was allowed for 4 h, and co-cultures were irradiated with 0 or 5 Gy plus 0–625 nM HSP90i treatment. After 48 h, cells were harvested by trypsinization, stained with 7-amino actinomycin D (7-AAD, BD Biosciences) for exclusion of non-viable phagocytes, and analyzed by flow cytometry. Phagocytosis was determined as percentage of 7-AAD negative, double-positive (PKH67, Hoechst) phagocytes. In order to confirm active prey cell engulfment, labeled DCs were pre-incubated with 10 μM cytochalasin D for 1 h before addition of the prey cells.

### Allogeneic Mixed Leukocyte Reaction

Allogeneic mixed leukocyte reactions (MLRs) were carried out as described previously (11). Briefly, naïve CFSE-stained T cells (positively selected on anti-CD3 magnetic beads) were added in a ratio of 10:1 to allogeneic human monocyte-derived DCs (1 × 10^4^ cells/well in 96-well plates) that had been differentiated in the presence of conditioned HCT116 culture supernatants in X-Vivo full medium. After 5 days, cells were collected, T cells were stained with anti-CD3-PE-Cy7, anti-CD4-PE, and anti-CD8-APC (all from BD Biosciences), and T cell proliferation was determined by flow cytometry based on the decline in T cell CFSE fluorescence intensity. Results are displayed as x-fold proliferation values normalized on the values obtained with HCT116 control supernatants.

### Purinergic P2Y Receptor RT-PCR

Purinergic P2Y receptor (P2RY) expression levels were determined by RT-PCR. Total RNA was isolated from THP-1 cells and primary human monocytes with the NucleoSpin RNA II Kit according to the manufacturer’s instructions, and reverse transcription was performed as described before (11). 10 ng of corresponding cDNA were subjected to amplification by PCR [10 min 95°C, 33x (15 s 95°C, 1 s 60°C)] with distinct primer pairs for each P2RY subtype (Supplementary Table 1). Human genomic DNA was used as positive and H_2_O as negative control. Amplification products were analyzed by agarose gel electrophoresis (3% agarose gel).

### Peritonitis Assays

*In vivo* recruitment of myeloid cells by culture supernatants of treated HCT116 cells was determined in peritonitis assays as described previously with minor variations (47–49). In brief, conditioned supernatants were injected intraperitoneally (*i.p.*) into C57BL/6 mice, and peritoneal lavages were collected from sacrificed mice after 6 h (*n* = 4 per group) or after 6, 12, and 24 h (*n* = 2 per group) for time course analyses, respectively. Total numbers of leukocytes were determined by using a Coulter A C T counter (Beckman Coulter, Krefeld, Germany). Cells were stained on ice for 30 min with the following monoclonal antibodies: anti-CD45-APC-Cy7 (from BD Biosciences), anti-CD11b-FITC, anti-Gr-1-PE, anti-F4/80-eFluor450 (from eBiosciences, San Diego, CA, United States). FACS analyses were performed upon lysis of erythrocytes (Gallios, Beckman Coulter). Monocytic cells and granulocytes were distinguished and quantified within the CD45^+^/CD11b^+^ myeloid cell population by cell surface expression levels of Gr-1 and F4/80.

### *M. cremaster* Assay and Intravital Microscopy

The single steps of leukocyte trafficking were examined in the *Musculus cremaster* assay by intravital microscopy as described before with minor modifications (48–51). Briefly, two groups of randomly assigned CX_3_CR1^*GFP/+*^ mice were injected intrascrotally (i.s.) and stimulated with conditioned supernatants for 6 h (*n* = 6 per group). After surgical preparation of the cremaster muscle, intravital microscopy was performed at an AxioTech-Vario 100 Microscope (Zeiss MicroImaging, Göttingen, Germany) equipped with a Colibiri LED light source (Zeiss MicroImaging) for fluorescence epi-illumination. Images were taken with an AxioCam Hsm digital camera with a 20 × water immersion lens (0.5 NA, Zeiss MicroImaging) and were processed with AxioVision 4.6 software (Zeiss MicroImaging). The region of interest (ROI) was analyzed with ImageJ (National Institutes of Health) for rolling, firm adhesion, and extravasation of leukocytes or CX_3_CR1^*GFP/+*^ monocytes, including classical (GFP^*low*^) and non-classical monocytes (GFP^*high*^), respectively. CX_3_CR1-expressing DCs were excluded from analyses on the basis of their characteristic stellate morphology.

### Statistical Analyses

Statistical analyses were performed using OriginPro 9.1 software (OriginLab Ltd., Northampton, MA, United States). 2-sided exact Wilcoxon Rank tests, or two-way ANOVAs were employed where indicated, and *post hoc* Bonferroni-Holm correction was applied where appropriate. Synergism was analyzed on the basis of combination indices (CIs) according to the Chou-Talalay Method (52). CIs indicate synergism (CI < 1), additivity (CI = 1), or antagonism (CI > 1).

## Results

### HSP90i Treatment Augments Radiation-Induced Death of Colorectal Cancer Cells and the Release of Monocyte Attracting DAMPs

**FIGURE 1:**
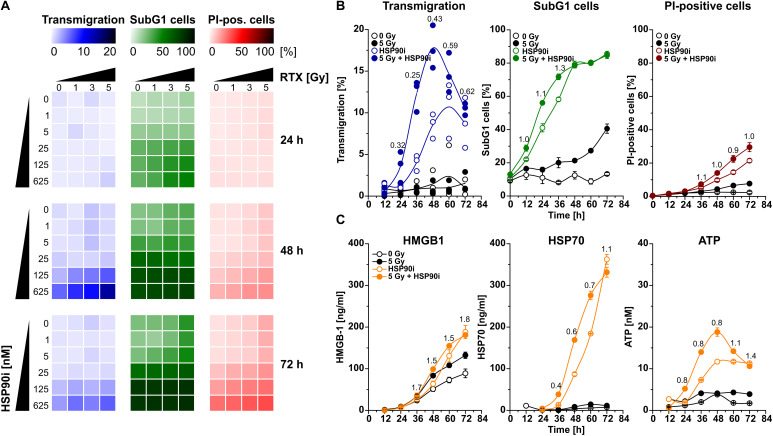
HSP90i treatment augments radiation-induced death of colorectal cancer cells and the release of monocyte attracting DAMPs. **(A)** Transwell migration of THP-1 cells toward cell-free supernatants of HCT116 cells (left row, blue), and induction of apoptosis and necrosis in HCT116 cells (middle row, green; right row, red). HCT116 cells were irradiated with 0–5 Gy in the presence of 0–625 nM HSP90i. Cell-free supernatants of HCT116 cells were harvested at the indicated time points and subjected to transwell migration assays with monocytic THP-1 cells. Induction of apoptosis and necrosis were measured by flow cytometry of subG1 nuclei (apoptotic HCT116 cells) and propidium iodide (PI) exclusion assay (necrotic HCT116 cells). Means of *n* = 3 independent experiments are shown. **(B)** Time course of migratory stimulus release, HCT116 cell apoptosis, and necrosis induction. HCT116 cells were irradiated at 5 Gy ± 625 nM HSP90i. Untreated HCT116 cells served as controls. Transwell migration assays and detection of apoptosis and necrosis were performed as in panel **(A)**. Combination indices (CIs) are depicted. CI values <1 indicate synergism between HSP90i and irradiation. Data points of *n* = 3 independent experiments (transwell migration) or means ± SD of triplicates of one representative experiment (apoptosis and necrosis) are shown. **(C)** Time course of DAMP release. HCT116 cells were treated as in panel **(B)**. Untreated HCT116 cells served as controls. Levels of HMGB1 (left panel), HSP70 (middle panel), and ATP (right panel) were quantified in cell-free supernatants 12–72 h after treatment by ELISA (HMGB1, HSP70) or luciferase assay (ATP), respectively. Means ± SD of triplicates of one representative experiment are shown, and CIs were calculated as in panel **(B)**.

An initial and essential step in the induction of anti-tumor immune responses is the attraction of monocytic cells and APC precursors by dying tumor cells (53, 54). For detailed analyses of the dynamics of cell death induction and monocyte attraction, HCT116 CRC cells were irradiated at doses of 0–5 Gy in the presence of 0–625 nM HSP90i. Monocyte attraction by cell-free supernatants was measured 24–72 h after irradiation *via* transwell migration assays with the monocytic cell line THP-1. In parallel, induction of apoptosis (determined as the percentage of cells with subG1 DNA content) and necrosis (determined as the percentage of cells with permeable plasma membrane) in treated HCT116 cells was examined by flow cytometry. HSP90i strongly increased irradiation-induced apoptosis and accelerated the transit into necrosis in a dose-dependent manner (Figure 1A). Supernatants of dying HCT116 cells stimulated a time- and dose-dependent migratory response in THP-1 cells with a maximum approximately 48 h after treatment. Comprehensive time course analyses confirmed that monocyte attraction was strongest with supernatants harvested 48 h upon treatment with 625 nM HSP90i and 5 Gy irradiation (Figure 1B). Therefore, 625 nM HSP90i + 5 Gy was chosen as standard combination treatment for further experiments. Notably, combination indices calculated according to Chou and Talalay (52) revealed a synergistic mode of action for HSP90i and irradiation (CI < 1) regarding the attraction of monocytic cells.

The release of DAMPs is a vital trigger for the attraction of monocytic cells by dying tumor cells (11, 42, 53, 54). Therefore, we measured the concentrations of several established DAMPs, including HMGB1, HSP70, and ATP, in culture supernatants of HCT116 cells upon treatment. The levels of HMGB1 and HSP70 increased over time especially in response to HSP90i and combined treatment with irradiation (Figure 1C). Interestingly, the release of ATP into the culture supernatants revealed similar kinetics as the migratory monocyte response in the transwell assays peaking 48 h after treatment with HSP90i plus irradiation (Figure 1B) and exhibited also the synergistic mode of action between HSP90i and irradiation. Taken together, in comparison to the monoagent settings, combination of HSP90i and irradiation results in synergistically enhanced DAMP release and monocyte attraction by dying tumor cells.

### DAMP Release and Monocyte Attraction Occur During Secondary Necrosis Upon Irradiation in Combination With HSP90i Treatment

**FIGURE 2:**
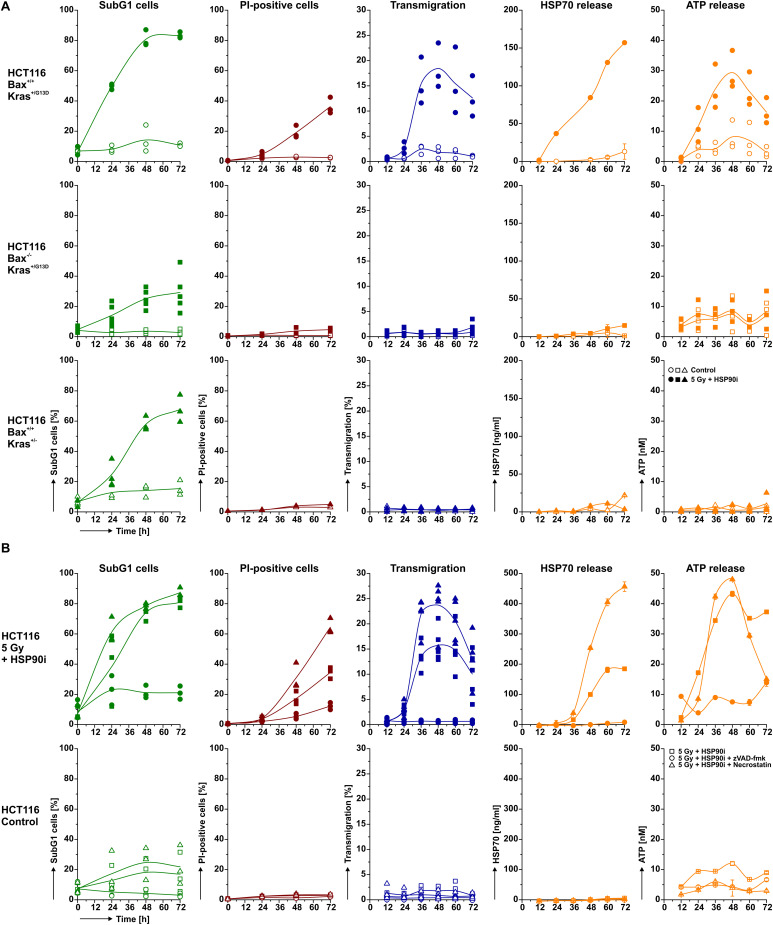
DAMP release and monocyte attraction occur during secondary necrosis upon irradiation in combination with HSP90i treatment. **(A)** Induction of cell death, THP-1 cell attraction, and DAMP release in HCT116 subclones with or without functional Bax and/or hyperactive Kras^*G*13*D*^. Parental HCT116 Kras^+/*G*13*D*^ Bax^+/+^ cells (upper row), HCT116 Kras^+/*G*13*D*^ Bax^–/–^ cells (middle row), and HCT116 Kras^+/–^ Bax^+/+^ cells (lower panel) were treated with 5 Gy plus 625 nM HSP90i or left untreated. At the indicated time points, induction of apoptosis (first column) and necrosis (second column), THP-1 cell transwell migration (third column), and release of HSP70 (fourth column) and ATP (last column) were measured as in Figure 1. Data points of *n* = 3–5 independent experiments (apoptosis, necrosis, transwell migration, ATP release) or means ± SD of triplicates of one representative experiment (HSP70 release) are shown. **(B)** Impact of different cell death inhibitors on apoptosis and necrosis induction, THP-1 cell attraction, and DAMP release in HCT116 cells upon treatment with radiotherapy and HSP90i. HCT116 cells were treated with 5 Gy plus 625 nM HSP90i in the presence of the poly-caspase inhibitor zVAD-fmk or the necroptosis inhibitor necrostatin-1, respectively (upper panel). Untreated cells served as controls (lower panel). Induction of apoptosis (first column) and necrosis (second column), THP-1 cell transwell migration (third column), and release of HSP70 (fourth column) and ATP (last column) were measured as in Figure 1. Data points of *n* = 3–5 independent experiments (apoptosis, necrosis, transwell migration) or means ± SD of triplicates of one representative experiment (release of HSP70 and ATP) are shown.

The mode of cell death is of central importance for the stimulationof immune responses. Particularly necrotic forms of cell death – primary as well as secondary, post-apoptotic necrosis – are known to be associated with the exposure of potent immunostimulating signals (5, 6, 55). In order to assess if necrosis induction and DAMP release are a common response of CRC cells to the combined treatment of HSP90i and irradiation, we made use of three additional CRC cell lines: HCT8, SW480, and HT29 cells. Whereas HCT8 cells showed a similar response pattern as HCT116 cells, yet with elevated amplitude, neither necrosis induction nor DAMP release were observed in HT29 cells (Supplementary Figure 1). SW480 cells revealed an intermediate response pattern. Despite the common colorectal origin, these cell lines differ in their mutational Kras status. While HCT116 and HCT8 cells are heterozygous for hyperactive Kras^*G*13*D*^, HT29 cells harbor two wildtype Kras alleles, and SW480 are homozygous for Kras^*G*12*V*^ which has been shown to be associated with moderate Kras activation (56, 57). Thus, our results point toward an involvement of hyperactive Kras^*G*13*D*^ in necrosis induction and DAMP release upon irradiation plus HSP90i treatment. However, since there are further molecular differences between these cell lines, we utilized isogenic subclones of HCT116 cells with genetically manipulated Kras status in order to characterize the mode(s) of cell death responsible for the observed release of monocyte attracting DAMPs in further detail. We also included HCT116 cells with manipulated Bax status to distinguish between apoptosis, primary, and secondary necrosis. So, apart from the established parental cell line HCT116 which is heterozygous for the activating Kras mutation G13D and has functional Bax, we employed HCT116 Kras^+/G13*D*^ Bax^–/–^ cells with hyperactive Kras lacking functional Bax (40) and HCT116 Kras^+/–^ Bax^+/+^ cells with both normal Bax and normal Kras function (41). All cell lines were treated with the combination therapy, and induction of apoptosis as well as necrosis, monocyte transwell migration, and DAMP release were monitored 0–72 h after treatment. In HCT116 parental cells, the combined treatment strongly induced apoptosis with subsequent transit into secondary necrosis, paralleled by robust monocyte attraction and release of HSP70 and ATP (Figure 2A, upper panel). HCT116 Kras^+/G13*D*^Bax^–/–^ cells, lacking the proapoptotic regulator Bax showed considerably reduced levels of apoptosis induction (Figure 2A, middle panel), supporting the common notion that ionizing irradiation and HSP90 inhibition stimulate apoptosis mainly *via* the intrinsic apoptosis pathway (37, 58, 59). Without preceding apoptosis, virtually no necrosis was observed in HCT116 Bax^–/–^ cells, indicating that the necrotic phenotype seen in treated parental HCT116 cells was in fact of the secondary, post-apoptotic kind. Cell-free supernatants of treated HCT116 Bax^–/–^ cells failed to attract monocytic cells, and only background levels of HSP70 and ATP were detected. In contrast to HCT116 Bax^–/–^ cells, HCT116 Kras^+/–^ Bax^+/+^ cells showed a strong induction of apoptosis upon treatment, comparable to the parental cells (Figure 2A, lower panel). However, apoptotic HCT116 Kras^+/–^ Bax^+/+^ cells did not transit into secondary necrosis, and neither monocyte attraction nor DAMP release were observed. These results clearly suggest that DAMP release and attraction of monocytic cells after combined treatment with HSP90i and irradiation occur in the phase of post-apoptotic, secondary necrosis.

In order to further prove the causal link between monocyte attraction, DAMP release, and secondary necrosis, we employed the poly-caspase inhibitor carbobenzoxy-valyl-alanyl-aspartyl-[O-methyl]-fluoromethylketone (zVAD-fmk) which interferes with apoptosis induction and necrostatin-1, an inhibitor of receptor-interacting serine/threonine-protein kinase 1 (RIPK1), which blocks necroptosis induction *via* RIPK1 (Figure 2B). Addition of zVAD-fmk clearly decreased apoptosis induction in parental HCT116 cells upon treatment, subsequently also preventing transit into secondary necrosis. Consequently, neither monocyte attraction nor DAMP release were observed. In contrast, treatment with necrostatin-1 did not impair but even increased necrosis induction, eventually resulting in elevated monocyte transwell migration and HSP70 release. In summary, these data underline the essential role of secondary necrosis for the release of monocyte attracting DAMPs by dying HCT116 cells upon combined treatment with HSP90i and irradiation. Analogous results obtained with HCT8 and SW480 cells further confirmed the post-apoptotic nature of necrosis induced by irradiation plus HSP90i treatment (Supplementary Figure 2).

### Monocyte Migration Inducing DAMPs Released Upon Irradiation Plus HSP90i Treatment Are Apyrase-Sensitive Nucleotides and Stimulate Chemokinesis *in vitro*

**FIGURE 3:**
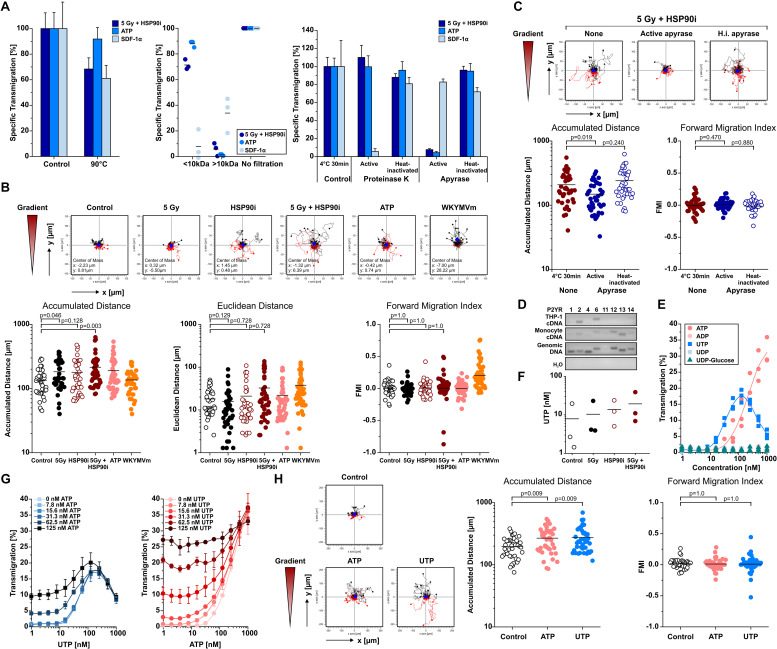
Monocyte migration inducing DAMPs released upon irradiation plus HSP90i treatment are apyrase-sensitive nucleotides and stimulate chemokinesis *in vitro.* **(A)** Biochemical characterization of monocyte attraction signals. HCT116 cells were treated with 5 Gy plus 625 nM HSP90i. 48 h after treatment, cell- and apoptotic body-free supernatants were subjected to heat-treatment (left panel), ultrafiltration (MW cut-off 10 kDa, middle panel), or enzymatic digestion with proteinase K or apyrase, respectively (right panel). The processed supernatants were subjected to THP-1 cell transwell migration assays. Culture media supplemented with ATP (200 nM) and SDF-1α (200 ng/ml) severed as controls. THP-1 transmigration results were normalized to the values of the non-treated supernatants. Data points of *n* = 3 independent experiments (ultrafiltration) or means ± SD of quadruplicates of one representative experiment (heat treatment, enzymatic digestion) are shown. **(B)** Monocyte chemotaxis/chemokinesis. HCT116 cells were treated with 5 Gy irradiation in the absence or presence of 625 nM HSP90i. Cell-free supernatants were subjected to 2D-chemotaxis analysis, and the migratory performance of primary human monocytes was monitored by live cell tracking for 3 h (analysis window 10–160 min). Cell-free supernatants from untreated HCT116 cells, ATP (1 μM), and WKYMVm (1 μg/ml) served as controls. Trajectory plots of 40 randomly picked primary human monocytes and their corresponding centers of mass (filled blue circles) are displayed. Accumulated distance, Euclidean distance, and forward migration index (FMI) were calculated. Bars indicate mean values of 40 cells. *p*-values were calculated by 2-sided exact Wilcoxon Rank test with *post hoc* Bonferroni-Holm correction. **(C)** Biochemical characterization of monocyte chemokinesis inducing signals. Cell-free supernatants of treated HCT116 (5 Gy + 625 nM HSP90i) were digested with apyrase as in panel **(A)**. Inactivated apyrase served as control. Chemokinesis of primary human monocytes induced by processed supernatants was analyzed as in panel **(B)**. **(D)** Expression of purinergic P2Y receptors (P2RY) in THP-1 cells and primary human monocytes. P2RY subtypes were amplified from THP-1 and primary human monocyte mRNA by RT-PCR, and PCR products were separated by agarose gel electrophoresis. Human genomic DNA was used as positive and ddH_2_O as negative control. **(E)** THP-1 cell transwell migration in response to different P2RY ligands. Transwell migration of THP-1 cells toward ATP, ADP, UTP, UDP, and UDP-glucose was measured in *n* = 3 independent experiments as in Figure 1A. **(F)** Release of UTP from HCT116 cells. HCT116 cells were irradiated with 5 Gy in the presence or absence of 625 nM HSP90i. 48 h after treatment, UTP levels were measured in cell-free supernatants by ELISA. Data points of *n* = 3 independent experiments are shown. **(G)** THP-1 cell transwell migration toward combinations of UTP and ATP. UTP (left panel) and ATP (right panel) were titrated at the indicated concentrations and THP-1 cell transwell migration was measured as in Figure 1A. Means ± SD of quadruplicates are displayed. **(H)** Induction of chemokinesis in primary human monocytes toward culture media supplemented with ATP (1 μM) and UTP (1 μM) was analyzed as in panel **(B)**. Non-supplemented culture medium served as control.

Necrotic cells release several DAMPs that have been reported to contribute to monocyte recruitment, including low molecular weight compounds such as nucleotides and high molecular weight compounds such as HSP70 and HMGB1 (5, 11, 42, 60). As shown in Figures 1, 2, the kinetics of ATP release upon treatment of HCT116 cells with HSP90i and irradiation closely paralleled the kinetics of monocyte transwell migration, thus pointing toward a crucial contribution of nucleotides to monocyte attraction in our model system. Nevertheless, other well-known DAMPs, such as HSP70 and HMGB1, were released at high concentrations as well. In order to dissect the involvement of different molecular classes of DAMPs, the monocyte attracting signals in supernatants of treated HCT116 cells were subjected to biochemical characterization experiments. As such, heat-treatment (90°C, 40 min), ultrafiltration (molecular weight cut-off ≤10 kDa), and enzymatic degradation (nucleoside triphosphate degrading apyrase or protein degrading proteinase K) were applied to culture supernatants of treated HCT116 cells before THP-1 cell transwell migration was analyzed (Figure 3A). ATP (MW = 507 Da) and the CXC chemokine stromal cell-derived factor 1 α (SDF-1α) (MW = 11 kDa) served as controls. In summary, the migration stimulating activity in culture supernatants of HCT116 cells treated with HSP90i and irradiation was observed to be largely heat stable with an apparent molecular weight ≤10 kDa, and sensitive to apyrase treatment. These results indicate that nucleotides released from secondary necrotic HCT116 cells are the key players in this scenario.

Nucleotides have been reported to induce undirected forms of migration (i.e., chemokinesis) and to act as auto- and paracrine amplifiers of other chemotactic stimuli rather than stimulating directed migratory responses in monocytic cells (i.e., chemotaxis) (42, 61, 62). We therefore characterized the mode of migration of primary human monocytes stimulated by supernatants of treated HCT116 cells by live cell imaging in 2D-migration chambers in greater detail (Figure 3B). The chemotaxis inducing formyl peptide receptor agonist WKYMVm and chemokinesis inducing ATP were used for comparison. In response to supernatants of HCT116 cells treated with HSP90i plus irradiation, monocyte migration was clearly increased but revealed an undirected pattern as indicated by the trajectory plots and quantified by significantly increased accumulated distance while Euclidean distance and forward migration index remained not significantly different from the controls. Notably, apyrase treatment of the culture supernatants completely abrogated the chemokinetic migration of monocytic cells, thus confirming the crucial role of nucleotides in our model (Figure 3C). Since apyrase hydrolyzes various nucleoside triphosphates to diphosphates and monophosphates (63), a more in-depth characterization of the monocyte attracting nucleotides released from treated HCT116 cells was performed.

Migration of monocytes in response to nucleotides is mediated by the family of P2Y receptors (P2RYs) (64). RT-PCR analyses of P2RY family members showed that THP-1 cells mainly express P2RY2 and P2RY6, whereas the spectrum in primary human monocytes was broader and included P2RY2, P2RY6, P2RY12, P2RY13, and P2RY14 (Figure 3D). The cognate ligands of these receptors were tested for their monocyte attracting potential. Only ATP and UTP, both ligands of P2RY2, were able to stimulate transwell migration of THP-1 cells and induced chemokinesis in primary human monocytes (Figures 3E,H). However, whereas the concentrations of purified nucleotides needed to induce THP-1 cell migration at comparable levels to supernatants of dying HCT116 cells ranged from 125 to 1000 nM for ATP and 30–250 nM for UTP, the measured concentrations of both nucleotides in the supernatants were only in the low nanomolar range (Figures 1, 2, 3F). To characterize potential interactions between ATP and UTP, monocyte attraction was examined in checkerboard titration experiments with different concentrations of both nucleotides. The combined effects of ATP and UTP on THP-1 cell migration showed additive behavior, without obvious synergism (CI ≈ 1). Still, with mixtures of ATP and UTP in concentrations analogous to the ones measured in the supernatants of treated HCT116 cells (ATP 20–40 nM and UTP 20 nM) THP-1 cell migration reached comparable levels (Figure 3G). Accordingly, we conclude that HSP90i plus irradiation stimulates the release of the P2RY2 ligands ATP and UTP by HCT116 cells which trigger monocyte transwell migration in a chemokinetic way *in vitro*.

### DAMPs Released Upon Irradiation Plus HSP90i Treatment Stimulate Myeloid Cell Recruitment *in vivo*

**FIGURE 4:**
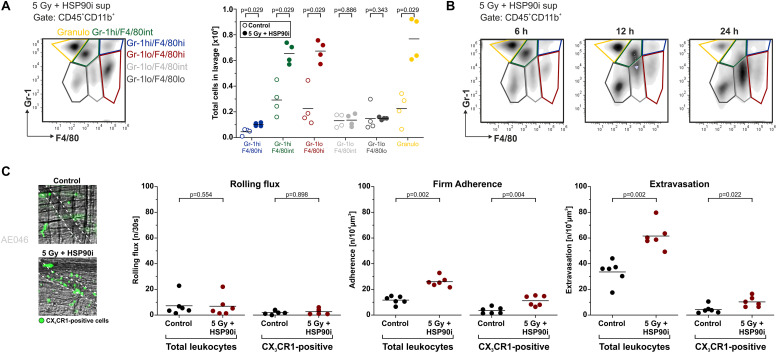
DAMPs released upon irradiation plus HSP90i treatment stimulate myeloid cell recruitment *in vivo*. **(A)** Recruitment of myeloid leukocyte subsets in the peritonitis model. Cell-free supernatants of treated HCT116 cells (5 Gy + 625 nM HSP90i) were harvested after 48 h and injected intraperitoneally (i.p.) into C57BL/6 mice. Supernatants from untreated HCT116 cells served as controls. 6 h after injection, the peritoneal lavage was collected, and myeloid leukocyte subsets were analyzed by flow cytometry. Representative density plots of peritoneal myeloid leukocytes (Gr-1 vs. F4/80 gated on CD45^+^CD11b^+^ cells) are shown. Quantification of peritoneal recruitment is depicted for classical monocytes (Gr-1^*hi*^F4/80^*hi*^, Gr-1^*hi*^F4/80^*int*^), macrophage-like cells (Gr-1^*low*^F4/80^*hi*^, Gr-1^*low*^F4/80^*int*^, Gr-1^*low*^F4/80^*low*^), and neutrophils (Gr-1^*hi*^F4/80^*low*^). Bars indicate median values of *n* = 4 mice per group. *p*-values were calculated by two-sided exact Wilcoxon Rank test. **(B)** Time course of myeloid cell recruitment. Recruitment of myeloid leukocyte subsets was analyzed 6, 12, and 24 h after supernatant injection as in panel **(A)**. Representative density plots of *n* = 2 animals are shown. Gray arrow indicates the shift in Gr-1 expression. **(C)** Extravasation of leukocytes in the *M. cremaster* model. Cell-free HCT116 cell supernatants were generated as in panel **(A)** and intrascrotally (i.s.) injected into CX_3_CR1^*GFP/+*^ mice. Leukocyte and CX_3_CR1^*GFP/+*^ monocyte trafficking in cremasteric venules were monitored by intravital microscopy 6 hours after injection. In representative images CX_3_CR1^*GFP/+*^ monocytes are depicted in green. Intravascular rolling, firm adherence, and extravasation was quantified for total leukocytes and CX_3_CR1^*GFP/+*^ monocytes. Bars indicate mean values of *n* = 6 mice per group. *p*-values were calculated by two-sided exact Wilcoxon Rank test.

*In vitro*, supernatants of HCT116 cells treated with HSP90i and irradiation stimulated undirected monocyte chemokinesis. However, for the priming of anti-tumor immune mechanisms directed recruitment of APCs and their precursors, such as monocytes, is crucial (65). To examine myeloid cell recruitment by dying tumor cell-derived DAMPs *in vivo*, we used the experimental peritonitis model, one of the standard model systems to assess leukocyte trafficking *in vivo*. Culture supernatants of HCT116 cells (treated with irradiation plus HSP90i or left untreated) were injected intraperitoneally into C57BL/6 mice, and after 6 h, recruited myeloid cell populations (CD45^+^CD11b^+^) were characterized by FACS analyses of the peritoneal lavage. In comparison to the untreated controls, supernatants of treated HCT116 cells stimulated significantly enhanced infiltration of different myeloid cell subsets. This applied to classical monocytes (Gr-1^*hi*^F4/80^*hi*^ and Gr-1^*hi*^F4/80^*int*^), macrophages (Gr-1^*low*^F4/80^*hi*^), and granulocytes (Gr-1^*hi*^F4/80^*low*^) (Figure 4A). Time course experiments also suggested that recruited classical monocytes subsequently start to differentiate into more macrophagocytic and/or APC-like phenotypes by downregulation of Gr-1 and upregulation of F4/80 (Figure 4B).

To further dissect the individual steps of myeloid cell recruitment, we made use of the *M. cremaster* model, a standard microcirculatory observation technique (66). Culture supernatants of HCT116 cells (treated with irradiation plus HSP90i or left untreated) were injected intrascrotally into CX_3_CR-1^*GFP/+*^ reporter mice. After 6 hours of stimulation, the trafficking of leukocytes and GFP-positive monocytic cells from postcapillary venules into the parenchyma of the *M. cremaster* tissue was monitored by intravital microscopy. Whereas no significant differences in the initial step of leukocyte rolling were observed, firm adhesion and extravasation were significantly increased in response to supernatants of HSP90i-treated and irradiated HCT116 cells as compared to the controls. These data indicate that the myeloid cell attracting potential of DAMPs released by dying HCT116 as observed *in vitro* also translates into increased transendothelial recruitment and tissue extravasation of different myeloid subsets *in vivo*.

### Differentiation and Effector Functions of Antigen Presenting Cells Are Enhanced Upon Contact With DAMPs Released From Irradiated and HSP90i-Treated Cancer Cells

**FIGURE 5:**
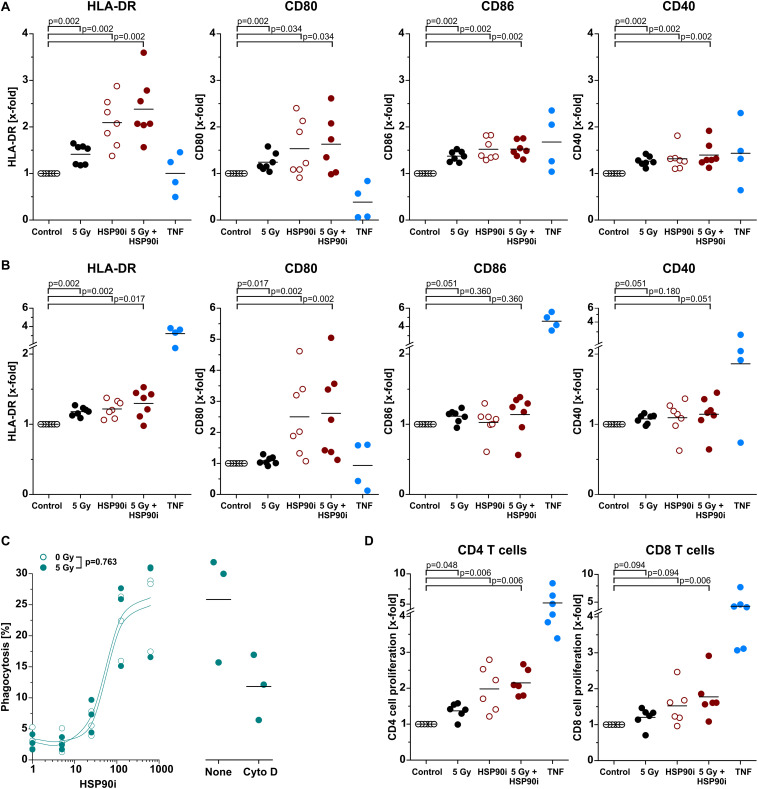
Differentiation and effector functions of antigen presenting cells are enhanced upon contact with DAMPs released from irradiated and HSP90i-treated cancer cells. **(A)** Differentiation of antigen presenting cells from primary human monocytes. HCT116 cells were treated with 5 Gy irradiation ± 625 nM HSP90i. Cell-free supernatants were harvested 48 h after treatment, and primary human monocytes were stimulated with the supernatants (1 + 1 in X-Vivo full medium) for 5 days. Surface expression of HLA-DR, CD80, CD86, and CD40 on monocytes were analyzed by flow cytometry. Supernatants from untreated HCT116 cells and TNF served as controls. Bars indicate mean values of *n* = 7 independent experiments. *p*-values were calculated by two-sided exact Wilcoxon Rank test with *post hoc* Bonferroni-Holm correction. **(B)** Differentiation of monocyte-derived dendritic cells. Primary human monocytes were differentiated in the presence of GM-CSF (20 ng/ml), IL-4 (40 ng/ml), and HCT116 supernatants (1 + 1 in X-Vivo full medium), and surface marker expression was analyzed as in panel **(A)**. Bars indicate mean values of *n* = 7 independent experiments. *p*-values were calculated by two-sided exact Wilcoxon Rank test with Bonferroni-Holm correction. **(C)** Phagocytosis of HCT116 cells by DCs. Co-cultures of PKH67-labeled DCs [differentiated for 4 days in the presence of GM-CSF (20 ng/ml) and IL-4 (40 ng/ml)] with Hoechst-labeled dying HCT116 cells (ratio 1:2) were treated with 0 or 5 Gy + 0–625 nM HSP90i. After 48 h, phagocytosis was determined by flow cytometry as the percentage of double-positive phagocytes (left panel). Datapoints for *n* = 3 independent experiments are shown. *p*-values were calculated by two-way ANOVA. Treatment with 10 μM cytochalasin D was used to confirm prey cell internalization (right panel). **(D)** T cell proliferation in allogeneic mixed leukocyte reactions (MLRs). Monocyte-derived DCs differentiated as in panel **(B)** were co-cultured with CFSE-labeled allogeneic CD3^+^ human blood T cells (ratio 1:10) for 5 days. The percentage of proliferating T cells was calculated as the percentage of CD3^+^CFSE^*low*^CD4^+^ or CD3^+^CFSE^*low*^CD8^+^ on the basis of all CD3^+^CD4^+^ or CD3^+^CD8^+^ cells, respectively. Bars indicate mean values of *n* = 6 independent experiments. *p*-values were calculated by two-sided exact Wilcoxon Rank test with Bonferroni-Holm correction.

Upon recruitment of monocytic cells, their differentiation into potent APCs is necessary in order to achieve T cell (cross-)priming for the stimulation of systemic, adaptive anti-tumor immune responses (4, 53). Along these lines, we next exposed primary human monocytes to cell-free supernatants of treated HCT116 cells and analyzed characteristic APC surface markers of the immunological synapse by flow cytometry after 5 days without any further differentiation stimulus. As such, the MHC class II molecule HLA-DR and the co-stimulatory ligands CD80 and CD86 as well as the co-activation marker CD40 were strongly upregulated, particularly upon exposure to supernatants of HCT116 cells treated with HSP90i and irradiation, indicating differentiation into an APC phenotype (Figure 5A). The most robust effects were observed for CD80 and HLA-DR, and similar findings were also obtained when IL-4 and GM-CSF were additionally supplemented in order to further support APC maturation (Figure 5B). For successful T cell (cross-)priming, tumor antigens need to be engulfed and processed by APCs. However, in contrast to APC surface marker expression, the engulfment of treated HCT116 cells by monocyte-derived dendritic cells was not affected by irradiation and only depended on the HSP90i concentration (Figure 5C). Finally, we analyzed the functional relevance of enhanced APC surface marker expression for their T cell (cross-)priming capacity by allogeneic MLR with CFSE-labeled primary T cells. Priming of both, CD4^+^ and CD8^+^ T cell proliferation was significantly enhanced by APCs differentiated in the presence of supernatants of HSP90i-treated and irradiated HCT116 cells. Collectively, our results indicate that DAMPs released by dying HCT116 cells upon irradiation plus HSP90i treatment support the recruitment, differentiation, and T cell (cross-)priming functions of APCs, and thus should favor the stimulation of systemic anti-tumor immune responses.

## Discussion

Despite its central role in the medical attendance of various cancer entities, indications of radiotherapy in CRC currently remain limited to malignancies of the rectum and high-risk cases of colon cancer receiving adjuvant fractionated (1.8–2 Gy per fraction) or neoadjuvant hypofractionated (5 Gy per fraction) radiotherapy alone or in combination with systemic chemotherapy, respectively (16, 17, 67). Due to the high mobility of the colon and the relevant radiosensitivity of the surrounding normal tissue, treatment planning, target volume delineation, and dose administration remain challenging, and supporting biological approaches for specific radiosensitization of the tumor appear highly attractive. In this regard, inhibition of HSP90 has been reported to be a promising strategy. Tumor cells show a strong dependence on HSP90 chaperoning function due to their high basal protein turnover and proteotoxic stress, and crucial regulators of the DNA damage response have been identified to be particularly susceptible to HSP90 inhibition (18, 19, 22, 27). Furthermore, HSP90 in tumor cells is largely organized in multi-chaperone complexes which exhibit higher affinity for HSP90i than “normal” HSP90 complexes in non-malignant cells, thus ensuring a relevant degree of tumor specificity for HSP90i-based targeted radiosensitization (68). In this context, we and others have previously shown the synergistic therapeutic efficacy of HSP90 inhibition in combination with radiotherapy in different models of CRC *in vitro* and *in vivo* (34, 37, 39).

Apart from targeted radiosensitization, HSP90 inhibition may also enhance the priming of anti-tumor immune mechanisms that has been observed upon radiotherapy but appears to be restricted to higher single doses and strongly hypofractionated protocols (3 × 8 Gy) (7–11, 42). Improved priming of anti-tumor immune mechanisms would be particularly desirable for patients with high-risk CRC whose tumors are prone to local failure as well as metastasis formation, and who receive radiotherapy in fractions of ≤ 5 Gy – for instance in combination with immunotherapeutic protocols (69–71). Therefore, the primary motivation for our present study was to examine how HSP90 inhibition enhances radiotherapy-induced immune cell priming with the clinical perspective to develop improved treatment options for high-risk cases of colon cancer. Here, we show that HSP90 inhibition in combination with radiotherapy induced apoptosis already at radiation doses ≤ 5 Gy in a Bax-dependent manner and accelerated transit into secondary necrosis *via* mechanisms involving hyperactive Kras in CRC cells. During secondary necrosis, dying tumor cells released different classes of DAMPs which stimulated migration and recruitment of monocytic cells *in vitro* and *in vivo* and induced differentiation of APCs with potent antigen presenting capacity. Thus, HSP90 inhibition obviously enables and supports the initial steps of anti-tumor immune priming upon radiotherapy at radiation doses ≤5 Gy.

Mechanistically, our results together with prior reports reveal that HSP90i-mediated apoptosis induction upon ionizing irradiation derives from targeted disintegration of crucial DNA damage response regulators and is executed *via* the intrinsic apoptosis pathway as indicated by its clear dependence on functional Bax (37, 39). The observed accelerated transit into secondary necrosis interestingly was dependent on hyperactive Kras. Whether this observation stems from the previously reported involvement of Kras in cytoskeletal rearrangements and cell softening (72), in mechanisms of autophagy (73, 74), in metabolic rewiring (75), or so far unknown functions of hyperactive Kras, respectively, requires further investigation. Nevertheless, since a relevant number of CRC cases exhibit oncogenic Kras mutations (16), the combination of HSP90 inhibition with radiotherapy seems particularly interesting for this subgroup of patients.

In the course of secondary necrosis, dying CRC cells released different classes of DAMPs – proteins, such as HMGB1 and HSP70, as well as nucleotides, including ATP and UTP. Whereas ATP and UTP increased the chemokinetic mobility of monocytic cells in a concerted way of action as similarly described in previous studies (11, 42, 61, 62), directed recruitment of myeloid cells *in vivo* may rather rely on protein DAMPs that activate endothelial cells and stimulate the upregulation of adhesion molecules and chemokines (11, 42). Along these lines, our detailed analyses of the recruitment process in postcapillary venules in the *M. cremaster* model revealed that predominantly the steps of firm leukocyte adherence and extravasation were facilitated by DAMPs released from dying CRC cells, whereas the preceding phase of leukocyte rolling remained largely unaffected (76). This kind of activation of vascular endothelial cells allowing improved immune cell recruitment has already been described for HSP90i monotherapy settings (77).

Among the populations of myeloid cells recruited by dying CRC cell-derived DAMPs in our *in vivo* experiments, classical monocytic cells (GR-1^*hi*^F4/80^*int–hi*^) appear to be of specific interest for the priming of anti-tumor immune mechanisms since they have been shown to prime tumor-specific CD8^+^ T cell responses *per se* or after intra-tumoral differentiation into potent APCs, respectively (78–80). The time-dependent decline in Gr-1 surface expression as observed in the experimental peritonitis model insinuated the differentiation into APCs (81) and was further confirmed in our *in vitro* differentiation experiments. Indeed, the upregulation of the MHC class II receptor HLA-DR, the co-stimulatory molecules CD80 as well as CD86, and the co-activating receptor CD40 upon stimulation of monocytes with supernatants of treated CRC cells indicates the emergence of a potent APC phenotype which was most pronounced upon exposure to supernatants of CRC cells treated with HSP90i plus radiotherapy. Functionally, this translated into significantly improved activation of allogeneic CD4^+^ as well as CD8^+^ T cells. Similar findings were obtained with supernatants of other human CRC cell lines upon irradiation with different fractionation protocols which stimulated activation of *in vitro* differentiated dendritic cells (82).

In our study, we focused on the initial steps of anti-tumor immune priming with particular interest in the release of dying cell-derived DAMPs upon radiotherapy and HSP90i treatment. Nevertheless, it should be noted that HSP90 inhibition has been described to enhance tumor immunogenicity on multiple other levels. As such, HSP90i treatment reportedly increases the expression of MHC class I and MHC class I-related molecules on tumor cells, enlarges the intracellular antigen pool, and improves the recognition of tumor cells by CD8^+^ T cells (83, 84). Further studies observed improved T cell-dependent killing of poorly immunogenic tumor cells by HSP90 inhibition *via* additional mechanisms involving inactivation of HER2/neu, EphA2, or TCLA1, respectively (85–87). Eventually also immune checkpoint inhibition has been reported to benefit from additional HSP90 inhibition (88). However, also contradictory results describing immunosuppressive effects of HSP90i have been published (89, 90). If the HSP90i-mediated immunological effects can add to the immunogenic reconditioning of the tumor microenvironment and the stimulation of anti-tumor immunity *in vivo* which have been observed in the context of radiotherapy (91) – ideally in a synergistic manner – requires more in-depth analyses. Apparently, the therapeutic success of such approaches in the clinical situation will depend on sequence and dose of both HSP90i and radiation (4, 19, 92).

In conclusion, our study shows that the therapeutic synergism between radiotherapy and HSP90 inhibition for the treatment of CRC is not only limited to the radiosensitizing effects of HSP90 inhibition but extends also to facilitated priming of anti-tumor immune mechanisms – specifically in case of Kras-driven tumors and at clinically relevant irradiation doses ≤ 5 Gy. However, further studies are needed in order to optimize treatment sequence and dose, and additional immune checkpoint inhibition might be considered with the aim of achieving the best therapeutic outcome for CRC patients.

## Data Availability Statement

The raw data supporting the conclusions of this article will be made available by the authors, without undue reservation.

## Ethics Statement

The studies involving human participants were reviewed and approved by Ethics Committee of the Medical Faculty of the LMU Munich. The patients/participants provided their written informed consent to participate in this study. The animal study was reviewed and approved by the Regierung von Oberbayern.

## Author Contributions

KL, CB, AE, RH, CR, BF, UG, NW, and MS conceived and designed the experiments. AE, RH, HK, GZ, BU, and JK performed the experiments and analyzed the data. NW provided the HSP90i NW457. SS and TS provided the HCT116 Kras^+/–^ Bax^+/+^ cells. AE, RH, NB, GZ, BU, and KL wrote the manuscript. All authors discussed the results and commented on the manuscript.

## Conflict of Interest

*epi*-pochoxime F (NW457) has been licensed by Nexgenix Pharmaceuticals (New York, NY, United States, acquired by Oncosynergy Inc.). NW has received funding from the company and has consulted for Nexgenix Pharmaceuticals. The remaining authors declare that the research was conducted in the absence of any commercial or financial relationships that could be construed as a potential conflict of interest.

## Abbreviations

7-AAD: 7-amino actinomycin D
APC: antigen presenting cell
AS: autologous serum
Bax: Bcl-2-associated X protein
CFSE: carboxyfluorescein diacetate succinimidyl ester
DAMP: damage-associated molecular pattern
DC: dendritic cell
DMSO: dimethyl sulfoxide
FCS: fetal calf serum
FMI: forward migration index
GM-CSF: granulocyte-macrophage colony-stimulating factor
HMGB1: high mobility group box1
HSP: heat shock protein
HSP90i: HSP90 inhibitor
i.p.: intraperitoneal
i.s.: intrascrotal
IFN: interferon
IL: interleukin
Kras: Kirsten Rat Sarcoma virus protein
MLR: mixed leukocyte reaction
PI: propidium iodide
PS: penicillin/streptomycin
SDF-1 α: stromal cell-derived factor 1 α
TLR: toll-like receptor
TNF: tumor necrosis factor
zVAD-fmk: carbobenzoxy-valyl-alanyl-aspartyl-[O-methyl]-fluoromethylketone.
