# Supplementary material for: Priming of Anti-tumor Immune Mechanisms by Radiotherapy Is Augmented by Inhibition of Heat Shock Protein 90
Source: Front Oncol. 2020 Aug 27;10:1668. doi: 10.3389/fonc.2020.01668 (PMC7481363; doi:10.3389/fonc.2020.01668)
Supplement: Supplementary file 1 [file Table_1.docx]

# **Supplemental Table**

| **Target** | **Forward primer** | **Reverse Primer** |
| --- | --- | --- |
| hP2RY1 | TCCGTGTACATGTTCAATTTGGC | AGTAGAAGATCAGGGCTGGCAGA |
| hP2RY2 | TCTACACCAACCTTTACTGCAGCA | TCGTAAGACGCCCAGACACC |
| hP2RY4 | CCCAACCCTATGGCTCTTCATC | GTGGAACATGTAGGTGGCCGT |
| hP2RY6 | CTACCGCGAGAACTTCAAGCA | TGGGTAATGACACAGATGTTCAGC |
| hP2RY11 | CACGGGAGCATGGC | GGAACCCACTGAGTTTGTCGTC |
| hP2RY12 | CACCAGTCTGTGCACCAGAGAC | CGCCAGGCCATTTGTGATA |
| hP2RY13 | CACCACAGTGATGCAAGGCTT | AGAGGGCTGGGAATACCAGCT |
| hP2RY14 | TCTAGCCGCAACATATTCAGCA | TCGGTCTGACTCTTTGTGTAGGG |

## **Supplemental Table S1:** Primer sequences of hP2RY PCRs.
