# Supplementary material for: Priming of Anti-tumor Immune Mechanisms by Radiotherapy Is Augmented by Inhibition of Heat Shock Protein 90
Source: Front Oncol. 2020 Aug 27;10:1668. doi: 10.3389/fonc.2020.01668 (PMC7481363; doi:10.3389/fonc.2020.01668)
Supplement: Supplementary file 2 [file Data_Sheet_1.docx]

# **Priming of anti-tumor immune mechanisms by radiotherapy is augmented by inhibition of heat shock protein 90**

Anne Ernst^1#*^, Roman Hennel^1*^, Julia Krombach^1^, Heidi Kapfhammer^1^, Nikko Brix^1^, Gabriele Zuchtriegel^2,3^, Bernd Uhl^2,3^, Christoph A. Reichel^2,3^, Benjamin Frey^4^, Udo S. Gaipl^4^, Nicolas Winssinger^5^, Senji Shirasawa^6^, Takehiko Sasazuki^7^, Markus Sperandio^3,8^, Claus Belka^1,9^, Kirsten Lauber^1,9+^

# **Supplemental Figures**

## **
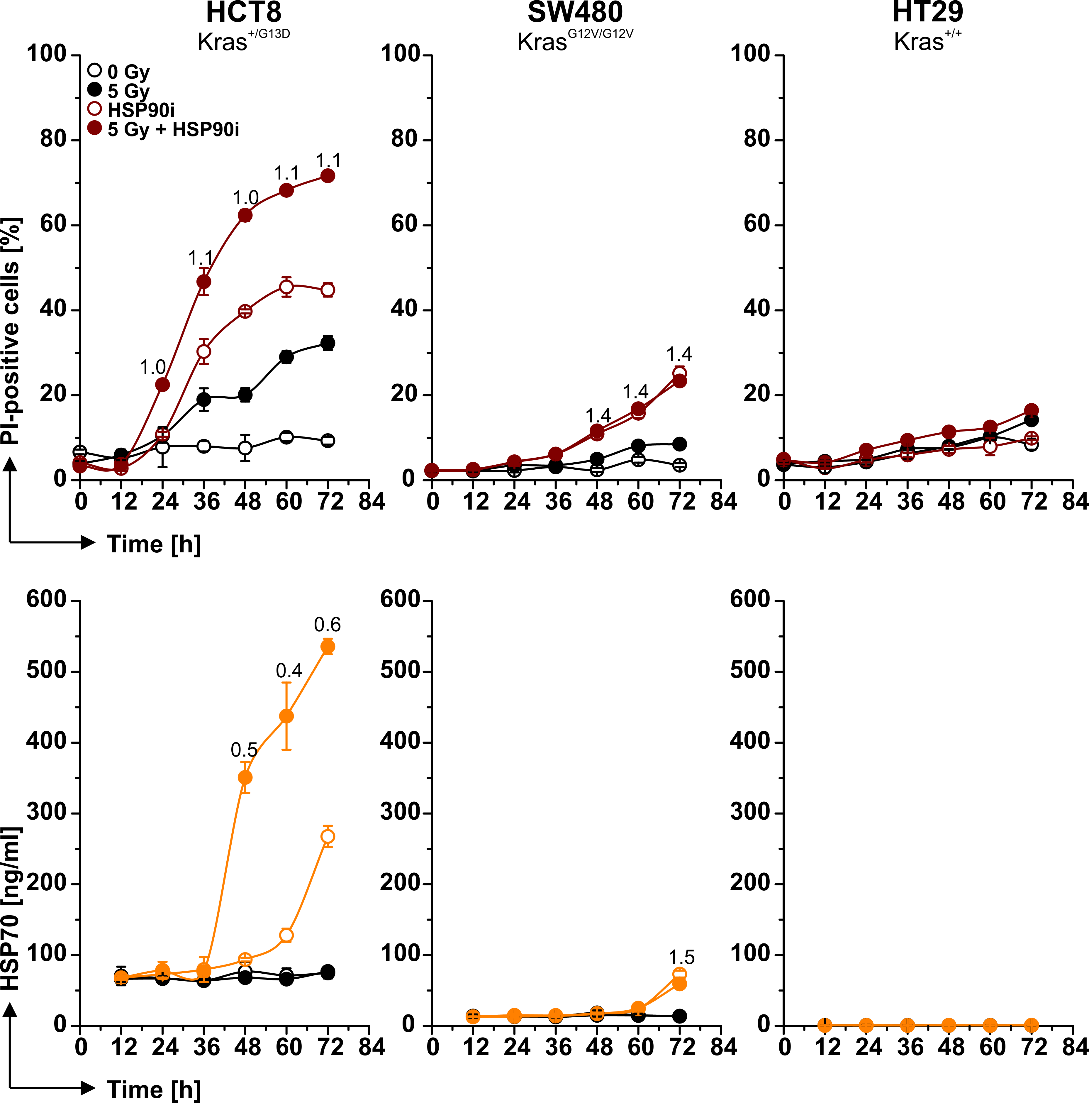
**

## **Suppl. Figure 1:** HSP90i treatment augments radiation-induced death of colorectal cancer cells and the release of HSP70.

Time course of necrosis induction and HSP70 release. HCT8, SW480, and HT29 cells were irradiated at 5 Gy ± 625 nM HSP90i. Untreated HCT116 cells served as controls. Detection of necrosis (upper panel) and HSP70 release (lower panel) were performed as in Figure 1. Combination indices (CIs) are depicted. CI values < 1 indicate synergism between HSP90i and irradiation. Means ± s.d. of triplicates of one representative experiment are shown.

## **
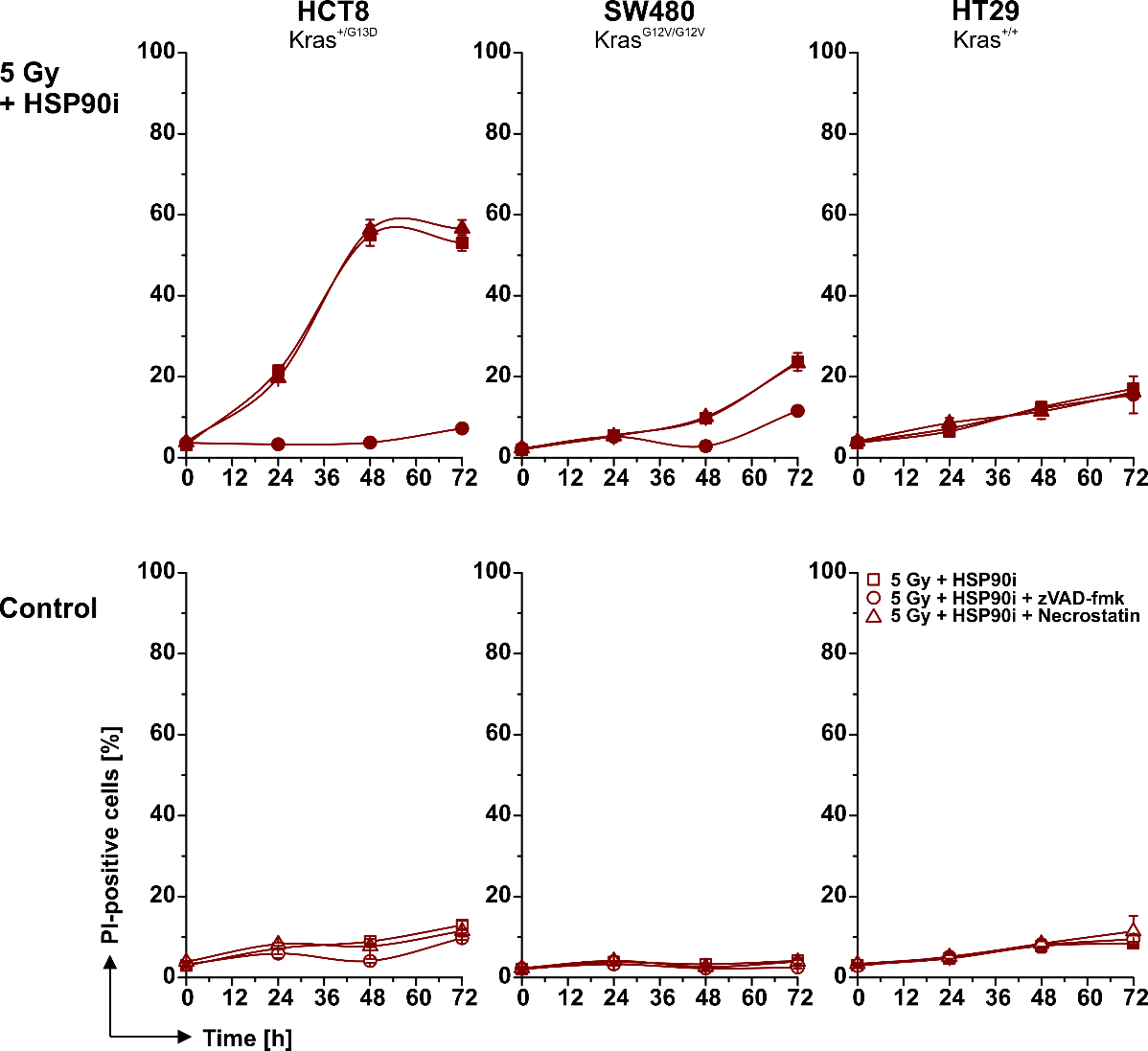
**

## **Suppl. Figure 2:** HSP90i treatment accelerates transit into secondary necrosis upon irradiation.

Impact of different cell death inhibitors on necrosis induction in colorectal cancer cells upon treatment with radiotherapy and HSP90i. HCT8, SW480, and HT29 cells were treated with 5 Gy plus 625 nM HSP90i in the presence of the poly-caspase inhibitor zVAD-fmk or the necroptosis inhibitor necrostatin-1, respectively (upper panel). Untreated cells served as controls (lower panel). Induction of necrosis was measured as in Figure 1. Means ± s.d. of triplicates of one representative experiment are shown.
